# Management of a Meniscus Tear in a Patient With Nail-Patella Syndrome: A Case Report

**DOI:** 10.7759/cureus.79461

**Published:** 2025-02-22

**Authors:** John Grossi, Lexi Garber, Montana Olexa, Kiana Zanganeh, Richard A Picerno II

**Affiliations:** 1 Research, Lake Erie College of Osteopathic Medicine, Bradenton, USA; 2 Osteopathic Medicine, Lake Erie College of Osteopathic Medicine, Bradenton, USA; 3 Orthopedic Surgery, Jacksonville Orthopaedic Institute, Jacksonville, USA

**Keywords:** degenerative joint disease, genetic syndromes, knee osteoarthritis/ koa, meniscus tear, nail patella syndrome

## Abstract

Nail-patella syndrome is a rare genetic condition that affects multiple organ systems and can lead to small or irregularly shaped patellae. Such irregular patellae can be of particular interest to orthopaedic practice because the small size can commonly lead to patella dislocation. Although the patient was initially evaluated for a medial meniscus tear, his underlying condition of nail-patella syndrome was found with incidental radiographs. The radiographic and MRI findings show hypoplastic patellae bilaterally with no dislocation or subluxation but with a medial meniscus tear. In this case report, we highlight the rarity of nail-patella syndrome, what to look for in the physical exams and diagnostic testing, and the importance of early and accurate diagnosis in order to increase awareness of the condition. While our patient did not present with patella dislocation, it was still crucial to address his underlying diagnosis of nail-patella syndrome. This anatomic variation guides the decision-making for treating concomitant orthopaedic issues such as a medial meniscus tear, as seen in our patient's case. In doing so, awareness of this genetic syndrome can affect future orthopaedic procedures such as knee replacements, and improve patient outcomes overall.

## Introduction

Nail-patella syndrome (NPS) is a genetic condition that affects the kidneys, nails, skeleton, and eyes [1]. NPS, also known as Fong disease, causes hypoplastic or dysplastic nails, absent or hypoplastic patellae, elbow deformities, and the presence of iliac horns [1]. This syndrome is typically transmitted in an autosomal dominant manner due to a mutation of the *LMX1B* gene on chromosome 9 [2].

NPS is very rare according to the National Organization of Rare Disease databases, occurring in one in 50,000 individuals [3]. Although nail changes are the most consistent feature amongst those who are affected, other common pathologies include glaucoma and renal failure, such as end-stage renal disease, hypertension, gastrointestinal (GI) involvement, and seizures [1]. In fact, often, the prognosis of NPS is based on renal function impact because 15-30% of those who have NPS have some renal involvement, and 15% of those will develop end-stage renal disease [4]. Commonly, the first sign of renal involvement is either hematuria or proteinuria [1]. From an orthopaedic aspect, the patellae will be hypoplastic but do not have to be symmetric in size [1]. The patellae can even be absent, and there can be prominent medial femoral condyles, hypoplastic lateral femoral condyles, and prominent tibial tuberosities [1]. 

The purpose of this report is to increase awareness of NPS as it can have various presentations. This report highlights the case of a patient who showed anatomical evidence of NPS on radiographic imaging; however, uniquely, he did not show clinical signs of patellar instability or dislocation. While the patient's primary concern for the visit was a medial meniscus tear, NPS was noted on imaging as well. Having an underlying diagnosis of NPS is rare and it is crucial for a clinician to be familiar with its anatomical variation, especially if these patients undergo any type of orthopaedic procedure. The small and irregular patellae can make orthopaedic surgeries more challenging and can impact the treatment of a medial meniscur tear.

## Case presentation

A 61-year-old male presented with left knee pain that started three months ago. The patient denied a specific trauma; however, he noticed the pain after he was helping move furniture. His pain was located on the medial aspect of the left knee and it was intermittent with certain movements such as standing and twisting the knee. Additionally, the patient reported that his knee felt unstable with activities such as walking and climbing stairs. His symptoms were worse when walking, sitting, and standing. He was taking non-steroidal anti-inflammatory drugs (NSAIDs), which improved his pain and inflammation. He described his pain as a dull ache. 

**Figure 1:**
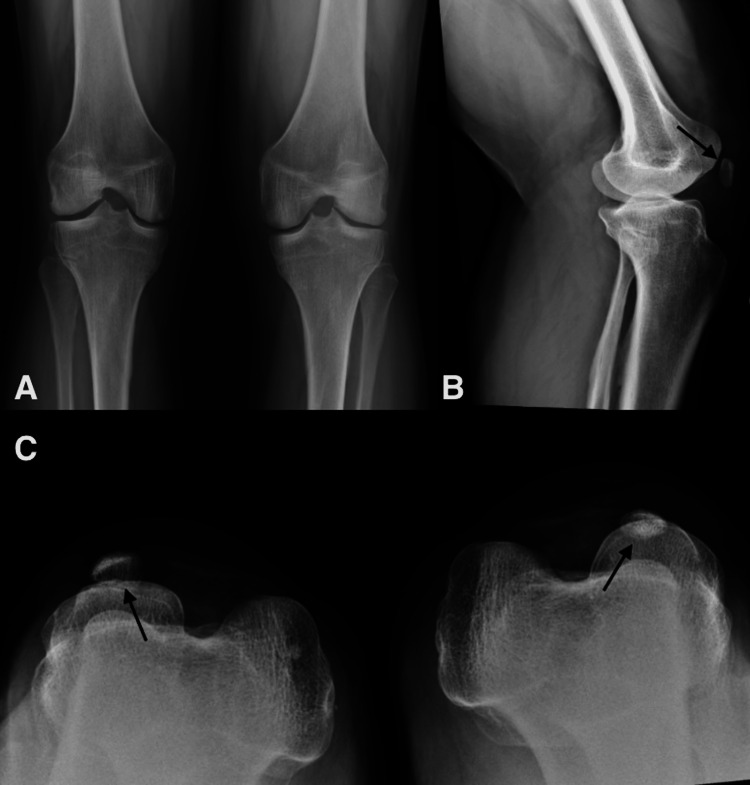
Anteroposterior (A), lateral (B), and sunrise (C) radiographs revealing bilateral tiny patellae (arrows)

On physical exam, he had an effusion of the left knee, with a range of motion of 0-130 degrees of flexion. He had tenderness along the medial aspect of the left knee, and a positive McMurray test, which was indicative of a meniscus tear. A Lachman exam, posterior drawer exam, and valgus/varus stress were negative, which ruled out ligamentous injuries. He had bilaterally absent patellae on exam. Anteroposterior, lateral, and patella sunrise radiographs (Figure 1) revealed very tiny patellas bilaterally, which was consistent with NPS. His radiographs revealed no significant degenerative changes, fractures, or dislocations with normal alignment. An MRI was ordered based on his history and physical exam being consistent with a medial meniscus tear. 

**Figure 2:**
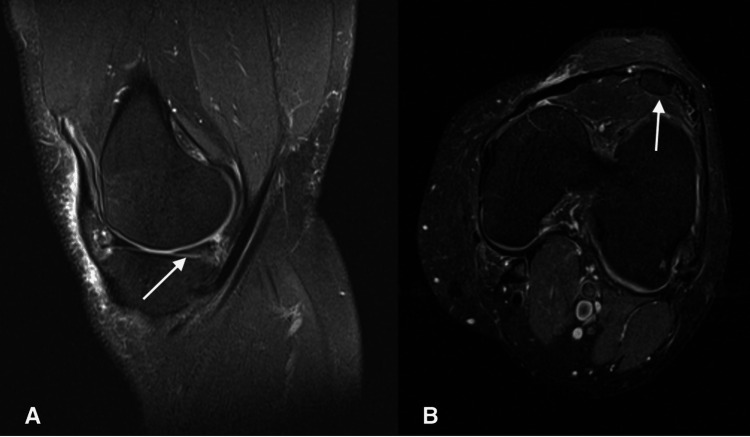
(A) Sagittal view of MRI reveal grade 2 intrasubstance posterior horn meniscus tear (arrow); (B) Axial view of the MRI reveal hypoplastic patella (arrow)

As shown in Figure 2, the MRI of the left knee revealed a grade 2 intrasubstance lesion of the posterior horn of the medial meniscus with a questionable radial tear of the free edge and multifocal full-thickness articular cartilage fissures of the posterior aspect of the lateral tibial plateau. Findings also revealed hypoplastic patella subluxed laterally without articular cartilage and absence of trochlea and trochlear articular cartilage, consistent with NPS. His symptoms improved at the time of the follow-up visit to the MRI. A corticosteroid injection with 2 cc of .25% Marcaine, 2 cc of 1% lidocaine, and 2 cc of Kenalog 40 mg/mL was injected into the left knee under sterile conditions to alleviate the patient's pain and inflammation. A knee arthroscopy with partial medial meniscectomy was considered if his symptoms persisted; however, the patient declined. 

## Discussion

The patient’s symptoms were concerning for a medial meniscus tear, given his intermittent medial knee pain, positive McMurray, and thus was confirmed with an MRI. In addition, on radiographic findings, bilateral hypoplastic patellae were noted, which raised concern for NPS. The patient also had hypoplastic nail changes on a physical exam, which further supported our diagnosis of NPS. Where most patients frequently experience knee instability and patellar dislocation, our patient did not. Thus, our patient’s case is unique because he did not exhibit any patellar instability issues. If the patient were to have patellar instability issues, there have been surgical treatments to realign the extensor mechanism with a medial patellofemoral ligament (MPFL) reconstruction [5]. If this patient had known about their diagnosis at an earlier age, surgical treatment with MPFL reconstruction could have been recommended to this patient in order to avoid early arthritis [6].

NPS has a unique pathology with a synovial band preventing the engagement of the patella into the trochlear groove, leading to contracture of the lateral soft tissues [6]. Therefore, when addressing the patella instability surgically, resection of the synovial band with additional realignment of the patella by recentering the quadriceps muscle is mandatory to avoid long-term morbidity [6].

While the patient did not have active symptoms related to his NPS, it was important to address his anatomical abnormalities to prevent future orthopedic issues. For instance, early diagnosis and treatment of his anomalies can prevent further degeneration of the articular cartilage of the knee and recurrent patellar subluxation [7]. If detected at a younger age, short-term relief with an arthroscopic retrograde lateral release with medial reefing can be performed. Although the current patient was older and did not have patellofemoral instability, it is important to keep this in mind when diagnosing other patients, especially younger patients with NPS in the pediatric population.

According to the MRI findings of the current patient, he showed the onset of degenerative changes, especially in the posterolateral compartment and trochlea. Therefore, it is possible that in the future this patient might need a total knee arthroplasty. In patients with NPS, there is no current gold standard of treatment of severe arthritis [5]. There have been many different strategies in treatment, including total knee arthroplasty without resurfacing the patella and unicompartmental patellofemoral arthroplasty [5]. Being that degenerative joint disease is common in the patient's age group and the current diagnosis of NPS, uniquely in his case, a different approach would be taken if or when he needs future orthopaedic surgery. 

Therefore, it is essential for orthopaedic surgeons to be aware of different approaches to surgical procedures, including total knee arthroplasty, in patients with NPS, as it can prevent unwanted revisions and poor outcomes. NPS is clinically relevant due to its rarity and anatomical variation, which can impact future orthopaedic surgeries and pathology such as degenerative joint disease. Additionally, the lack of literature on meniscal tear treatment in patients with NPS should prompt an increase in research to help further develop a standardized treatment plan.

## Conclusions

NPS is a rare and unique diagnosis that clinicians should keep in mind when diagnosing their patients. Our patient had a unique presentation where he showed anatomical evidence of NPS but did not show clinical signs of patellar instability specifically. Although the primary concern for our patient's visit was a medial meniscus tear, his underlying NPS should prompt clinicians to consider different approaches for surgical management. The anatomic variation in NPS guides a unique approach for current and future orthopaedic surgeries, as standard procedures can be more challenging in these patients due to the smaller and irregular patellae. Early and accurate diagnosis of NPS can allow for earlier preventative measures and tailored treatment plans, to facilitate better patient outcomes.
